# COVID-19 health information system assessments in eight European countries: identified gaps, best practices and recommendations

**DOI:** 10.1093/eurpub/ckae041

**Published:** 2024-07-01

**Authors:** Miriam Saso, Nienke Schutte, Marika Borg, Neville Calleja, Andrea E Schmidt, Mariana Peyroteo, Luís Velez Lapão, Angela Fehr, Martin Thißen, Michael Courtney, Petronille Bogaert

**Affiliations:** Health Information System Unit, Scientific Directorate of Epidemiology and Public Health, Sciensano, Brussels, Belgium; Health Information System Unit, Scientific Directorate of Epidemiology and Public Health, Sciensano, Brussels, Belgium; Ministry for Health, Valletta, Malta; Ministry for Health, Valletta, Malta; Department of Climate & Health, Austrian National Public Health Institute (GÖG), Vienna, Austria; CHRC, NOVA Medical School, Faculdade de Ciências Médicas, NMS, FCM, Universidade Nova de Lisboa, Lisboa, Portugal; UNIDEMI, Department of Mechanical and Industrial Engineering, NOVA School of Science and Technology, Universidade NOVA de Lisboa, Caparica, Portugal; Laboratório Associado de Sistemas Inteligentes, LASI, Guimarães, Portugal; CHRC, NOVA Medical School, Faculdade de Ciências Médicas, NMS, FCM, Universidade Nova de Lisboa, Lisboa, Portugal; UNIDEMI, Department of Mechanical and Industrial Engineering, NOVA School of Science and Technology, Universidade NOVA de Lisboa, Caparica, Portugal; Laboratório Associado de Sistemas Inteligentes, LASI, Guimarães, Portugal; WHO Collaborating Center on Health Workforce Policy and Planning, Instituto de Higiene e Medicina Tropical, Universidade NOVA de Lisboa, Lisboa, Portugal; Centre for International Health Protection, Robert Koch Institute, Berlin, Germany; Department for Health Monitoring and Epidemiology, Robert Koch Institute, Berlin, Germany; Department of Health, Dublin, Ireland; Health Information System Unit, Scientific Directorate of Epidemiology and Public Health, Sciensano, Brussels, Belgium

## Abstract

**Background:**

Global threats, such as the coronavirus disease 2019 (COVID-19) pandemic, have highlighted the critical importance of robust and well-functioning health information systems (HIS) in effectively addressing public health emergencies. To enhance the understanding and the functioning of such systems, it is crucial to perform HIS assessments. This article explores key gaps and identifies best practices in the COVID-19 HIS of eight European countries. Furthermore, it provides recommendations to strengthen European systems for better pandemic preparedness.

**Methods:**

Assessments were carried out in eight European countries using an adapted version of the WHO support tool to strengthen HIS and the Joint Action on Health Information assessment tool. The assessments took place between January 2022 and April 2023.

**Results:**

Four main themes emerged regarding the gaps and best practices identified in the various HIS: organizational, technical, legal and resources. The results of these assessments show different approaches implemented by countries to improve their HIS and respond to the demands of the pandemic.

**Conclusions:**

It is imperative for countries to draw valuable insights from the COVID-19 pandemic and strengthen their HIS. This involves the adaptation or development of pandemic preparedness plans, strengthening legislative framework for data sharing and privacy protection, promotion of data standards and international definitions and implementation of a unique person identifier. Additionally, countries will have to act in this post-pandemic era and integrate the newly developed systems and innovations into existing structures, maintain and develop trust by citizens through transparent communication and engage in infodemic management and address resource gaps in the workforce.

## Introduction

Health information systems (HIS) play a pivotal role in the preparedness, response and management of pandemics. These systems serve as the backbone for collecting, analysing and disseminating essential health data and information to inform evidence-based decision-making.^1^ Global health threats, such as the recent coronavirus disease 2019 (COVID-19) pandemic, have highlighted the critical importance of robust and well-functioning HIS in effectively addressing public health emergencies.^2–4^ European countries exhibit distinct and diverse HIS, shaped by their unique historical and socio-cultural context. While there have been notable advancements in HIS across Europe, studies have revealed that progress is not uniform among countries.^5^ Such differences have been exacerbated by the COVID-19 pandemic which has pushed countries to rapidly adapt their HIS to identify, collect, store, manage and share COVID-19 data in a timely manner,^4^ both in a narrow sense on the population’s health status and service coverage directly, as well as indirectly on the determinants of population health during the pandemic.^2^^,^^3^

In order to improve the understanding and the functioning of HIS, it is crucial to assess HIS.^6^ Such assessments provide valuable insights into the strengths and weaknesses of information systems, helping to identify gaps and bottlenecks that may hinder the timely and accurate collection, management and utilization of health data. Additionally, they serve as a foundation for designing targeted interventions to strengthen HIS and identify best practices that can be implemented by other systems.^6^ Previous research has partially shown that some European countries were able to capitalize on investments performed prior to the pandemic, while others had to drastically improve or develop new systems to meet the pandemic’s requirements.^3^^,^^7^ At the same time, the pressure experienced by the different HIS across Europe brought innovation and yielded valuable lessons regarding both best practices and areas requiring improvement.^8^

Here, we showcase the results of COVID-19 HIS assessments aimed to map national HIS and their capacity to respond to the effects of COVID-19 on population health in eight European countries. This allows the identification of existing gaps and best practices. Furthermore, this study contributes to the existing body of research by providing recommendations to strengthen national HIS in preparation of future health crises that may challenge the resilience of these systems.

## Methods

This study was performed within the framework of PHIRI, the Population Health Information Research Infrastructure,^9^ which aims to facilitate and generate the best available evidence for research on health and well-being of populations as impacted by COVID-19.

### Assessment schedule

COVID-19 HIS assessments were performed (January 2022 to April 2023) through 90 expert interviews in eight countries across Europe: Italy, Portugal, Ireland, Malta, Norway, Hungary, The Netherlands and Belgium (table 1). The selection of the countries was based on the responses to a call of interest among PHIRI’s consortium partners.

**Table 1:** Stakeholders interviewed during the COVID-19 health information system assessments in the different countries chronologically ordered

Country	Stakeholders interviewed	Department/service/Team/Unit
Italy	Italian National Institute of Health (ISS)	Department of Cardiovascular, Endocrine-metabolic Diseases and Aging
	Italian Office for Statistics (ISTAT)	Integrated Health, Assistance, Social security & Justice Service Population Registry, Demographic Statistics and Living Conditions Service
	Ministry of Health (MoH)	Directorate General for Digitization, Health Information System and Statistics
	Italian National Institute of Health (ISS)	Department of Infectious Diseases
	Sicilian Region	Scientific Technical Committee (CTS) for the COVID-19 Pandemic
	Presidency of the Council of Ministers	Communications Office
Portugal	National Institute of Health (INSA)	Department of Epidemiology
	Directorate-General of Health of Portugal (DGS)	Information and Analysis Services Department
	Shared Services of the Ministry of Health (SPMS)	Central Administration of Health
	National Statistics Institute (INE)	Department of Management and Financing of Health Benefits
Ireland	Central Statistics Office (CSO)	Statistical System Coordination Unit
	Health Service Executive (HSE)	Quality and Patient Safety Directorate Mental Health Division Health Intelligence Unit
	Health Information and Quality Authority (HIQA)	Health Information and Standards Directorate
	Department of Health (DoH)	Statistics and Analytics Unit
	National Office of Clinical Audit (NOCA)	National ICU Audit Team Information Management Team
	Health Protection Surveillance Centre (HPSC)	Epidemiology and Surveillance Team
Malta	Ministry for Health (MFH)	Superintendence of Public Health COVID-19 Public Health Response Team Infectious Disease Prevention and Control Unit Severe Acute Respiratory Infections (SARI) Team Directorate for Health Information and Research
	University of Malta	Public Health Department
	Hekk Hu (volunteers)	Communication team
Norway	Norwegian Directorate of Health	Division of Health Data and Digitalisation Division of Health Intelligence and Policy Global Health and Health Intelligence Department Legal Department Specialized Health Care Department
	Norwegian Institute of Public Health	Infection Control Registries Department Health Services Research Department Vaccine and Preventable Diseases Department Infection Control and Preparedness Department
Hungary	National Public Health Center (NNK)	Epidemiology of Communicable Diseases Department
	Health Services Management Training Centre Semmelweis University	Health Service Department
	National e-Health Infrastructure (EESZT)	Operations Management Team
	National Directorate General for Hospitals (OFKO)	Directorate for Project Coordination
Netherlands	National Institute for Health and Environment (RIVM)	Epidemiology Department Signalling and Surveillance Department National Coordination of Infectious Disease Control Directorate Vaccination Programme COVID behavioural Unit
	Ministry of Health, Welfare and Sport	COVID Directorate
	Association of Regional Public Health Services and Regional Medical Emergency Preparedness and Planning (GGD GHOR)	Monitoring & COVID-19 Information Team
	National Intensive Care Evaluation (NICE)	Quality Registry Team
	ZonMw	Infectious Diseases Programme Team
Belgium	Sciensano (National Public Health and Research Institute)	Crisis Coordination Service Epidemiology of Infectious Diseases Service Health Information Service Health Services Research Service Healthcare-Associated Infections and Antimicrobial Resistance Healthdata.be Service
	National Institute for Sickness and Invalidity Insurance (RIZIV/INAMI)	Health Care Service
	eHealth Platform	
	Catholic University Leuven	Biostatistics and Statistical Bioinformatics Centre
	Hasselt University	Foresight and Scenario Modelling Team

**Figure 1:**
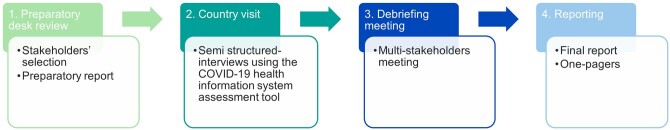
Schematic representation of the four steps of a COVID-19 health information system assessment as employed in the current study

The methodology employed for the assessments was based on the country visits performed in the Joint Action on Health Information (InfAct, HP-JA-2017, 801553) and the one delineated in the *Support tool to assess health information systems and develop and strengthen health information strategies* by the WHO Regional Office for Europe.^10^^,^^11^Figure 1 shows schematically the four steps of a COVID-19 HIS assessment. A detailed explanation of the presented steps can be found in the PHIRI HIS assessments manual.^12^

The assessment procedure included three main roles.

- The assessor: serving as an impartial and professional evaluator conducting the assessment within the assigned country.
- The contact person: the national liaison throughout the assessment process who identifies the key stakeholders, organizes and coordinates the interviews.
- The facilitator: to ensure continuity throughout the different assessments, one or multiple facilitators from Sciensano (Belgium) and/or the Ministry for Health (Malta) participated in all the interviews.

Assessors and contact persons were briefed and trained in a two-day course on how to perform the COVID-19 HIS assessment in October 2021.

### Tool

The assessment tool was derived from the S*upport tool to assess health information systems and develop and strengthen health information strategies* by the WHO Regional Office for Europe.^11^ The tool was updated in 2021, to better reflect the context of the evolving HIS in the WHO European Region, especially regarding digital evolution and knowledge translation. The core module was adapted to reflect this need, and it now comprises a comprehensive set of items associated with data collection, analysis, reporting, knowledge translation and governance and resources in a HIS. For each item, accompanying guiding questions designed to assist the assessor, along with a description that provides a benchmark for how the situation should ideally function within a fully developed HIS, are provided. Additionally, several add-on modules were added to the core module, aiming to zoom in on specific parts or functions of the national HIS.^13^ For this exercise, the add-on module on infectious disease surveillance was added to the core module and adapted to be able to map COVID-19 HIS (Supplementary table S1). Table 2 shows the overview of domains and sub-domains covered in the final assessment item list used in this study.

**Table 2:** Overview of domains and sub-domains covered in the assessment item list

Core domains	Sub-domains
Data collection	Data sources Data infrastructure Data management Resources
Analysis	Data analysis Indicators Big data and artificial intelligence (AI) Resources
Reporting	Health reporting Policy integration Resources
Knowledge translation	Information and knowledge products Resources
Governance and resources	Legislation Policy, planning and evaluation Resources

### Data collection and reporting

The preparatory report “for each assessment” (step 1 in figure 1) was developed by the assessors based on documentation (and related websites) compiled by the contact persons. The preparatory report provided key background information on the COVID-19 HIS of the country and facilitated the identification of the stakeholders for the interviews. Subsequently, semi-structured interviews were organized using the COVID-19 HIS assessment item list (step 2). The interviews were not recorded; however, detailed notes were taken by the assessors and facilitators during all the interviews. The assessors and facilitators met halfway through the interviews and after the interviews in order to draft a strengths, weaknesses, opportunities and threats (SWOT) analysis, in which the elements were included after consensus was achieved. An initial assessment report was presented at the multi-stakeholder meeting (step 3). For this meeting, all the interviewed stakeholders were invited to provide initial feedback on the findings. An assessment report was compiled by the assessors (step 4) and revised by the facilitators and the contact persons. This final report, included a SWOT analysis, specific, measurable, achievable, relevant and time-bound recommendations and a one-pager summarizing the main findings of the assessments, was then shared with the interviewed stakeholders. All the one pagers were published on the European Health Information Portal,^14^ while the full-detailed country reports were not made available in the public domain and remain confidential for the use of the country assessed.

For the purpose of this publication, the SWOT analyses that were generated from each assessment were pooled and screened by the assessors and the facilitators. Sequentially, through organized discussion meetings, recurring themes were identified and discussed among the facilitators and the assessors. Finally, overarching outcomes across the assessments were summarized and categorized according to the identified themes and recommendations for potential improvement on gaps and best practices of COVID-19 HIS were formulated.

## Results

From the SWOT analyses performed in each assessment, four main themes emerged with regards to the best practices and gaps identified in the various HIS (table 3).

**Table 3:** Overview of identified gaps and best practices in assessed European countries’ health information systems

Theme	Identified gaps	Best practices
Organizational	Use of paper-based records and manual data entry Low collaboration between the public and the private health sector Absence of an infodemic management strategy Inconsistent sharing of information to the citizens Absence of a well-established network for exchanging information within the country and at the European level	Involvement of the private sector beyond health Involvement of experts in an interdisciplinary manner for communication Development of targeted interventions to reach vulnerable or less responsive sub-set of the population Set up of clear data dashboards Opening of new communication channels to answer citizens’ concerns Regular publishing of public reports
Technical	Lack of interoperability between health systems at the institute, regional or national level Absence of a consistently used unique personal identifier Inconsistent use of definitions and international standards	Linkage of databases for the identification of vulnerable groups Expertise to quickly set up technological surveillance systems Establishment of a COVID-19 data lake containing data depersonalized in a standard manner
Legal	Lack of sufficiently developed pandemic preparedness plans, requiring emergency legislation for pandemic data flows Lack of long-term monitoring and surveillance strategies Lack of plans on how to integrate the newly developed systems with the ones already in place Variations in the interpretation of the general data protection regulation (GDPR)	Investments in strengthening health information systems (HIS) prior the pandemic Interdisciplinary expert board as part of the pandemic preparedness plan
Resources	Systematic lack of human resources Prolonged and challenging hiring processes Absence or limited availability of training opportunities for the health personnel	Reallocation of personnel to support urgent public health task Involvement of students and volunteers to mitigate the shortage of specialized human resources Involvement of universities to benefit from their expertise Participation to European Projects to design and implement HIS courses

### Organizational

With regards to organization aspects, the first identified gap was the use of paper-based records and manual data entry. During the pandemic, such practices limited accessibility to patient information and decreased interoperability, making it challenging to exchange data between different systems. According to the stakeholders, this approach led to inefficient record management, as healthcare providers had to manually input the same information across multiple systems, resulting in time-consuming and error-prone activities. Additionally, paper-based records lacked real-time updates, which negatively impacted both patient care and health research initiatives.

The second identified gap pertains to the limited collaboration between public and private health providers that resulted in data gaps. During the pandemic, private testing centres were collecting data on COVID-19 cases; however, in certain countries, there were no legal obligations or formal agreements in place that obliged the private centres to share the data with the research or public health institute. The stakeholders reported that this led to incomplete data as not all COVID-19 cases were counted in the national statistics. On the contrary, some countries reported the existence of smooth collaborations when collecting data from e.g. private hospitals and testing centres. Additionally, some countries initiated innovative collaborations with the private sector that extended beyond health to learn from their expertise. These include supply chain knowledge that could be used for efficient vaccine delivery, temporary transfer of personnel from inactive services (e.g. aviation services) to support public health tasks and collaborations with the telecommunication sector to measure and monitor the effectiveness of physical distancing measures using aggregated mobile phone data.^15^

A third identified gap pertains to communication to the public. In the absence of an effective communication and infodemic strategy, the stakeholders highlighted that a rapid dissemination of false and harmful information was recorded, potentially eroding trust in health authorities. On the contrary, in countries where these aspects were appropriately addressed, a higher level of trust in health authorities was registered in the citizens with positive uptake and compliance to public health measures.^16^ Additionally, the involvement of experts in an interdisciplinary manner, the setup of clear data dashboards, the development of tailored campaigns, the opening of new channels such as free emergency phone numbers to answer citizens’ concerns and regular publishing of public reports allowed for better knowledge translation and facilitated the uptake of health information by the citizens.

Another gap that was identified relates to the absence of a well-established network or platform to answer urgent public health questions during the crisis within and between countries. The stakeholders emphasized that this absence hindered the exchange of information, effective coordination and communication among health stakeholders, ultimately leading to sub-optimal decision-making during the crisis.

### Technical

Within this theme, the first identified gap was the lack of interoperability between health systems at the institute, regional and national level. The stakeholders reported that this caused limited or slower data exchange between stakeholders, and it increased the need for technical support to successfully allow systems to communicate. When such support was not available, stakeholders had to perform manual data extraction and transfer processes, which can be time-consuming, costly, prone to mistakes and may increase the risk of unauthorized data access. On the contrary, in other countries, interoperability across systems was supported and linkage of databases was performed, allowing for richer insights and the identification of vulnerable groups, e.g. for vaccination delivery. Countries also demonstrated quick reaction times and where interoperable systems were not available, new surveillance systems were set up, including digital contact tracing systems, allowing for the collection of COVID-19 data.

The second identified gap relates to the absence of a consistently used unique personal identifier. This hindered the secure and efficient exchange of patient information, creating missed opportunities for data linkage and resulting in the duplication of health records. A best practice identified in this regard was the work performed in certain countries to establish a COVID-19 data lake containing data that are depersonalized in a standard manner, supporting the linkage of databases.

The third key identified gap was the inconsistent use of definitions and international standards causing inconsistency in terminology, case definition, classifications, incomparability of data with other countries and decreased interoperability between systems hindering collaboration, data exchange and decision-making during the crisis.

### Legal

Within this theme, the first identified gap was the lack of sufficiently developed pandemic preparedness plans. Certain countries lacked clear guidelines on communication and coordination between different stakeholders, resulting in fragmented efforts and duplication of work, delays in decision-making and poor resource allocation leading to staff and equipment shortages. The stakeholders reported that this pushed countries to develop emergency legislations to set up new data flows, on how to share data across key health players and clarify the roles of the different stakeholders in the country. A best practice was the availability of national plans to assemble an interdisciplinary board of experts to promote the quick development of strategies to tackle different aspects of the pandemic. Additionally, the decision of certain countries to invest in strengthening their systems before the pandemic allowed them to capitalize on their investment. Strong fully digitalized surveillance systems updated before the crisis, supporting different automated data processes such as data validation and data quality checks, facilitated quick implementation of new data flows and more accurate results.

The second key identified gap was the lack of long-term monitoring and surveillance strategies. As many COVID-19 data flows were initiated as part of the emergency legislation, a lot of concern was raised on their long-term use and the possibility of such data being destroyed after the emergency status in a country was withdrawn, resulting in the loss of valuable information on the behaviour of the virus and insights on population health. Additionally, countries lacked plans on how to integrate the newly developed systems with the ones already in place to improve data collection on other respiratory diseases and capitalize on the investments done during the pandemic.

Another gap was the national interpretation of the general data protection regulation (GDPR) and, subsequently, its effect on national legislative frameworks. During the pandemic, the interpretation of key terms such as ‘public interest’ and ‘personal data’ caused a lot of discussion across health stakeholders and led to considerably different interpretations across Europe. Some stakeholders highlighted that the rise of concerns from citizens and the opening of legal cases on the potential risks associated with the sharing of their personal data led to stricter interpretations of the GDPR and the implementation of more stringent consent requirements. In countries where the GDPR was interpreted more strictly, health professionals had to obtain consent from each patient to use their data for research in a manner called ‘opt in system’. With individuals needing to actively opt in, participation rates may be low, limiting the generalizability of the findings as the sample may not be representative of the entire population. On the contrary, some countries interpreted the GDPR in the ‘public interest’ perspective. Under this interpretation, the GDPR recognizes that safeguarding public health and addressing a public health emergency can justify the processing of personal data without explicit consent, as long as appropriate safeguards are in place.^17^

### Resources

Related to resources, the first identified gap was the systematic lack of human resources, especially with technical IT skills, at all levels in the health sector. The SWOT analysis highlighted how such shortage hindered the development and implementation of effective COVID-19 response plans while causing overburden on the limited specialists available. Additionally, certain countries experienced long bureaucratic hiring processes, exacerbating the burden on the health personnel. A best practice identified in this regard was the flexibility demonstrated by some stakeholders in reallocating personnel at the national level to support urgent public health endeavours. This approach allowed for the optimization of available human resources, ensuring a swifter response to the evolving demands of the pandemic. Furthermore, the SWOT analysis showed that there has been a proactive engagement of universities to leverage their expertise and collaborate with national public health institutes. This collaboration has facilitated the development of advanced statistical models and supported data analysis to address critical information requests.

The second identified gap relates to the absence or limited availability of specific training opportunities for the health workforce. With the development of new systems and the shift towards more digitized operations, health personnel may lack the necessary knowledge and skills to effectively align with technology innovation and demand. A best practice observed within this theme was the engagement of students and volunteers to mitigate the shortage of specialized human resources which was supported by health stakeholders, including universities, with the provision of guidance and tailored training. Additionally, most countries participated in European Projects to design and implement HIS courses oriented to address urgent COVID-19 issues and strengthen the next generation of public health information professionals in Europe.

## Discussion

Following the results of the assessments, several overarching recommendations can be formulated. First, countries need to be encouraged to revise or develop their preparedness plans and include elements related to the strengthening of their HIS. This entails, among others, investments in surveillance systems for early detection and monitoring of diseases, guidance on response to threats procedures and coordination mechanisms for collaboration.^18^^,^^19^

A second recommendation relates to the establishment of an adequate legislative framework that facilitates data sharing and supports the re-use of health data. This aligns with the current developments related to the European Health Data Space (EHDS) and the need for countries to prepare for its implementation to support the cross-border use of health data.^20^^,^^21^ The results of the current study highlight how European countries need to address their shortcomings to meet the EHDS requirements. This includes the implementation of (meta)data standards, use of international definitions, data quality labels and the adoption of a unique person identifier to facilitate the exchange and linking of data, enabling comprehensive insights and cross-border collaboration. The EHDS has the potential to ensure countries are prepared for the next health challenge in terms of timely exchange of high-quality health data and information, which lies at the basis of the generation of sound scientific evidence to underpin decision-making.

Taking into consideration the challenges of a post-pandemic era, another recommendation pertains to finding sustainable solutions for integrating the newly developed systems and the innovations brought by the pandemic in existing structures to be used and adapted for future emergencies. With the progressive decrease of attention on COVID-19, countries need to act quickly as they may lose the momentum to concretely strengthen their HIS. In this regard, the adoption of an international agreement focused on pandemic prevention and preparedness under the WHO can empower countries worldwide to strengthen capacities to address and mitigate future pandemics. The adoption of such an international instrument could encourage a higher and sustained level of political engagement by European and world leaders, placing the issue on the agenda of heads of states and governments. Secondly, this instrument could establish clear processes and responsibilities, limiting ambiguity and streamlining international cooperation in times of crisis. Finally, this instrument could promote the integration of health considerations into all relevant policy areas, emphasizing that global health is not solely a medical issue but an essential component of overall global stability and prosperity.^22^ Furthermore, working on the trust and confidence of citizens in health authorities is an essential aspect of post-pandemic time. Countries are recommended to maintain or strengthen citizens’ trust by prioritizing transparency, clear communication, and active engagement with the public.^16^^,^^23^ Countries are encouraged to develop infodemic strategies including the proactive dissemination of accurate and reliable information, leveraging various communication channels, and effectively countering misinformation through targeted education campaigns and fact-checking initiatives.^24^

Finally, addressing the resource gaps was identified as a paramount concern.^25^ Countries are recommended to invest in human resources, particularly IT profiles in healthcare, to enhance the technical expertise required for the development, implementation, and maintenance of robust HIS. To systematically address such shortages, countries are encouraged to collaborate with universities to strengthen and increase availability of courses on data management. Additionally, training healthcare professionals in digital skills and promoting a culture of data-driven decision-making are highlighted as essential steps in strengthening the overall capacity of the healthcare workforce.^26^ In this regard, the Digital Education Action Plan (2021–27) with its strategic priorities and planned actions may play a key role in supporting the development of a high-quality digital education ecosystem and digital skills and competences for the digital transformation our societies are facing.^27^

Some limitations could be identified in this study. Firstly, the selection procedure was based on PHIRI's consortium partners’ availability which may have inadvertently excluded countries lacking resources or experience to participate. To limit this risk, the partners were informed of the support the facilitators would provide throughout the assessment. Secondly, different assessors and stakeholders interviewed may have varying knowledge, biases or interpretations, which can introduce subjectivity and affect the consistency of the assessments. To address this limitation, assessors were trained on how to carry HIS assessments and were supported by the facilitators to increase consistency across the assessments. Furthermore, multiple stakeholders have been interviewed in each country to obtain a more well-rounded opinion on the country’s HIS. Thirdly, depending on the time of the assessment, the roles in a COVID-19 management team might have shifted, missing the opportunity to provide insights on the decisions taken prior to this change. Fourthly, the reticence of some national experts in sharing shortcomings of their national HIS due to political sensitivities, fear of repercussions, or a desire to maintain a positive image could have limited the capacity of the assessment to capture the full range of challenges and gaps within the HIS. To address this limitation, the interviews were not recorded and the detailed reports were not published publicly. Finally, due to the COVID-19 pandemic, the interviews had to be performed online. On one side, this limited the sharing of experiences while on the other, it allowed the contact persons to reach a wider number of experts due to the flexibility in planning the interviews.

## Supplementary Material

ckae041_Supplementary_Data

## Data Availability

For each country assessed a detailed report containing findings from the interviews was prepared. These reports were shared among the national stakeholders that took part to the interviews but are not publicly accessible. The decision to share the complete report remains at the discretion of the stakeholders interviewed within the country of interest. One-page summaries for multiple countries can be found on the PHIRI website at https://www.phiri.eu/covid-19-his-assessments. For more information, please contact the corresponding author. Key points By implementing lessons learned from the COVID-19 pandemic, countries can enhance the resilience, efficiency and responsiveness of their health information infrastructures, thereby bolstering their ability to effectively address future health threats. Stronger health information systems will require the adaptation or development of pandemic preparedness plans, strengthening of the legislative framework for data sharing and privacy protection, promotion of data standards and international definitions and implementation of a unique person identifier. Countries will have to act in this post-pandemic era and integrate the newly developed systems and innovations into existing structures for future emergencies, maintain and strengthen trust of citizens through transparent communication and engage in infodemic management. Addressing resource gaps through investments in IT profiles, training healthcare professionals in digital skills, and promoting data-driven decision-making will be vital for strengthening overall healthcare capacity and preparedness.

## References

[1] Marieke V , Oers van H. *Population Health Monitoring: Climbing the Information Pyramid*. Springer International Publishing, 2019. Available at: http://link.springer.com/10.1007/978-3-319-76562-4 (May 2023, date last accessed).

[2] Azzopardi-Muscat N , Kluge HHP, Asma S, Novillo-Ortiz D. A call to strengthen data in response to COVID-19 and beyond. J Am Med Inform Assoc 2021;28:638–9. 33275146 10.1093/jamia/ocaa308 PMC7798979

[3] Schmidt AE , Abboud LA, Bogaert P. Making the case for strong health information systems during a pandemic and beyond. Arch Public Health 2021;79:13–6. 33514433 10.1186/s13690-021-00531-5 PMC7844779

[4] Liu C. Health information systems amid COVID-19 outbreak: lessons from China. Health Inf Manag 2021;50:99–100. 32806959 10.1177/1833358320947557

[5] Bogaert P , Verschuuren M, Van Oyen H, Van Oers H. Identifying common enablers and barriers in European health information systems. Health Policy 2021;125:1517–26. 34666917 10.1016/j.healthpol.2021.09.006

[6] Noël R , Taramasco C, Márquez G. Standards, processes, and tools used to evaluate the quality of health information systems: systematic literature review. J Med Internet Res 2022;24:e26577. 35258469 10.2196/26577 PMC8941431

[7] Negro-Calduch E , Azzopardi-Muscat N, Nitzan D, et al Health information systems in the COVID-19 pandemic: a short survey of experiences and lessons learned from the European region. Front Public Health 2021;9:676838. 34650946 10.3389/fpubh.2021.676838 PMC8505771

[8] Thomas S , Sagan A, Larkin J, et al Strengthening health systems resilience: key concepts and strategies, 2020. https://eurohealthobservatory.who.int/publications/i/strengthening-health-system-resilience-key-concepts-and-strategies (June 2023, date last accessed). 32716618

[9] Sciensano. The Population Health Information Research Infrastructure (PHIRI). Available at: https://www.phiri.eu/ (26 May 2023, date last accessed).

[10] Bogaert P , Verschuuren M, Abboud L, et al Assessing European national health information systems in peer review format: lessons learnt. Eur J Public Health 2023;33:580–4. 37263589 10.1093/eurpub/ckad085 PMC10395761

[11] WHO. Support tool to assess health information systems and develop and strengthen health information strategies, 2015. https://iris.who.int/handle/10665/172761 (May 2023, date last accessed).

[12] Schutte N , Saso M, Borg M, Bogaert P. PHIRI Health Information System (HIS) assessments manual version 3.0. Zenodo 2022:1–24. 10.5281/zenodo.6951897.

[13] WHO. Support tool to strengthen health information systems: guidance for health information system assessment and strategy development, 2021.

[14] Sciensano. Health Information System Assessments: Health Information Portal. Available at: https://www.healthinformationportal.eu/services/health-information-system-assessments (June 2023, date last accessed).

[15] Szocska M , Pollner P, Schiszler I, et al Countrywide population movement monitoring using mobile devices generated (big) data during the COVID-19 crisis. Sci Rep 2021;11:5943. 33723282 10.1038/s41598-021-81873-6 PMC7961025

[16] OECD. Building Trust to Reinforce Democracy: main findings from the 2021 OECD Survey on Drivers of Trust in Public Institutions. OECD, 2022. (Building Trust in Public Institutions). Available at: https://www.oecd-ilibrary.org/governance/building-trust-to-reinforce-democracy_b407f99c-en (18 August 2023, date last accessed).

[17] European Parliament, Council of the European Union. Regulation (EU) 2016/679 of the European Parliament and of the Council of 27 April 2016 on the protection of natural persons with regard to the processing of personal data and on the free movement of such data, and repealing Directive 95/46/EC (General Data Protection Regulation), 2016. Available at: https://eur-lex.europa.eu/legal-content/en/TXT/?uri=CELEX:32016R0679&qid=1694788827758 (June 2023, date last accessed).

[18] ECDC. Lessons from the COVID-19 pandemic. Stockholm, 2023. Available at: https://www.ecdc.europa.eu/en/publications-data/lessons-covid-19-pandemic-may-2023 (June 2023, date last accessed).

[19] Morgan OW , Aguilera X, Ammon A, et al Disease surveillance for the COVID-19 era: time for bold changes. Lancet 2021;397:2317–9. 34000258 10.1016/S0140-6736(21)01096-5 PMC8121493

[20] European Commission, Directorate-General for Health and Food Safety. Proposal for a regulation of the Euroepan Parliament and of the Council on the European Health Data Space. Available at: https://eur-lex.europa.eu/legal-content/EN/TXT/?uri=CELEX%3A52022PC0197 (15 September 2023, date last accessed).

[21] Abboud L , Cosgrove S, Kesisoglou I. Country Factsheets—Mapping Health Data Management Systems through Country Visits: Development, Needs and Expectations of the EHDS. Brussels: Sciensano, 2023.

[22] European Council. An international agreement on pandemic prevention and preparedness. Available at: https://www.consilium.europa.eu/en/policies/coronavirus/pandemic-treaty/ (9 January 2023, date last accessed).

[23] Mansoor M. Citizens’ trust in government as a function of good governance and government agency’s provision of quality information on social media during COVID-19. Gov Inf Q 2021;38:101597. 34642542 10.1016/j.giq.2021.101597 PMC8494525

[24] WHO. An ad hoc WHO technical consultation managing the COVID-19 infodemic: call for action. Geneva: WHO, 2020. Available at: https://www.who.int/publications/i/item/9789240010314.

[25] Merkur S , Maresso A, Cylus J, et al Lessons from the first wave: the COVID-19 Health System Response Monitor (HSPM) an evidence resource and a source of analysis. Eurohealth 2020;26:5–9.

[26] Haldane V , De Foo C, Abdalla SM, et al Health systems resilience in managing the COVID-19 pandemic: lessons from 28 countries. Nat Med 2021;27:964–80. 34002090 10.1038/s41591-021-01381-y

[27] European Commission, Directorate-General for Education, Youth, Sport and Culture. Communication from the Commission to the European Parliament, the Council, the European Economic and Social Committee and the Committee of the Regions—Digital Education Action Plan 2021-2027 Resetting education and training for the digital age. European Union: European Commission, 2020. Available at: https://eur-lex.europa.eu/legal-content/EN/TXT/PDF/?uri=CELEX:52020DC0624 (June 2023, date last accessed).

