# Supplementary material for: COVID-19 health information system assessments in eight European countries: identified gaps, best practices and recommendations
Source: Eur J Public Health. 2024 Jul 1;34(Suppl 1):i74–80. doi: 10.1093/eurpub/ckae041 (PMC11215322; doi:10.1093/eurpub/ckae041)
Supplement: ckae041_Supplementary_Data [file ckae041_supplementary_data.pdf]

Supplementary table I: COVID-19 health information system assessment item list adapted from the item list of the WHO Support tool to strengthen health information systems

Item ID	Item ID_WHO	Question	Probing question	Expectations
**Data sources**				
Data sources_1	InfDisSurv_1	Does the country have any infectious disease/pathogen surveillance system in place?	(1) Please describe the infectious disease/pathogen surveillance system in place. What is the mechanism for identifying notifiable infectious diseases and reporting them to the relevant authorities?	(1) The country has nationwide mandatory and regular passive notification of priority infectious diseases. In addition, active sentinel surveillance is conducted for selected infectious diseases targeted for eradication or elimination (such as measles). The surveillance system must cover 80% of level 3 administrative units in the country to be considered “nationwide”.
			(2) Please describe sources used for infectious disease surveillance and mechanisms of collecting data (paper, fax, electronic, phone). If no electronic reporting systems for infectious diseases exist, are there plans to implement electronic reporting in the future?	(2) Infectious disease surveillance reports are submitted from physicians at the community and primary care level, hospitals, laboratories and private health care facilities. The country has an interoperable, interconnected electronic reporting system in place, which is sustained by the government. Laboratory data are reported electronically (from reporting laboratories or laboratory networks).
			(3) What is the proportion of reporting sources, at subnational and local levels, with a standardized reporting system?	(3) The proportion of subnational/local reporting facilities with a standardized reporting system is high. The surveillance system should cover 80% of level 3 administrative units in the country to be considered “nationwide”.
			(4) Is a national database COVID-19 registry in operation?	(4) An infectious diseases database on COVID-19 that facilitates storage, management, extraction, querying and sharing of data among stakeholders is in place. Laboratory data are received electronically (from reporting laboratories or laboratory networks) and linked to epidemiological and clinical data.
			(5) Does the country conduct sentinel surveillance?	(5) Examples are given of sentinel surveillance system in place (e.g. for influenza-like illness and severe acute respiratory infections).
			(6) Regarding COVID-19, are any special arrangements in place to cover high-risk yet hard-to-reach populations/settings such as prisons and refugee camps? Please provide examples.	(6) Where routine notification procedures are not feasible, are disease surveillance programmes for COVID-19 in place, such as syndromic surveillance, that specifically target high-risk, vulnerable and hard-to-reach populations. Examples of such programmes are provided.
			(7) Is there a GIS database integrated with the infectious disease surveillance system? Please list practical examples of the different uses of GIS resources within the ministry of health or other agencies and partners.	(7) Concrete examples of GIS systems and their uses are given (e.g. assessing spatial distribution of a disease /mapping, monitoring results of disease surveillance, planning/targeting interventions).
Data sources_2	Data sources_10	What data sources from other domains are available?	(1) Are innovative methods, state-of-the-art alghoritms and digital tools being used for COVID-19 health monitoring?	(1) Innovative methods, state-of-the-art alghoritms and digital tools are being used for COVID-19 health monitoring, including social media, devices and AI.
			(2) What type of population health data sources are at your disposal on the wider impacts of COVID-19?	(2) Various data sources from other domains that are relevant for population health in relation to COVID-19 are available, such as social security data and data on, for example, the (mental) health of students from schools and universities, retirement statistics, injuries/accidents, crime records, road accidents, air pollution, ambient noise, the living environment (e.g. green spaces, walkability), threats related to food safety and threats of chemical or radiologic/nuclear origin.
Data sources_3	Data sources_2	What is the status of registration of vital statistics - related to COVID-19 and its wider effects?	(1) Are any COVID-19 deaths unregistered and, if so, what share of deaths – and which subgroups of the population – does this concern?	(1) The coverage of COVID-19 registered deaths is (close to) 100%.
			(2) What is the coverage of cause-of-death information recorded on the death registration form?	(2) The coverage of cause-of-death information recorded on the death registration form is (close to) 100%
			(3) What is the quality of the cause-of-death information recorded on the death registration form related to COVID-19?	(3) Cause-of-death information recorded on the death registration form related to COVID-19 is of high quality:<br>- medical doctors are trained (as part of the regular curriculum and/or postgraduate training) to fill in the death registration forms;<br>- clear rules and a legal framework that define under which circumstances an autopsy needs to be performed to establishing the cause of death are in place;<br>- the International Statistical Classification of Diseases and Related Health Problems, 10th revision (ICD-10) is used to code causes of death;<br>- coding is performed by dedicated, specifically trained staff;<br>- the proportion of all deaths coded to ill-defined causes is low<br>- COVID-19 death certificates are regularly matched and updated
Data sources_4	Data sources_3	What is the status of health service records related to COVID-19?	(1) What kind of record-keeping systems are used in hospitals and in primary health care/by general practitioners? How are these records compiled and how is the information shared, with regards to COVID-19?	(1) A centralized electronic health record (EHR) system is in place. If various electronic information systems are used, interoperability between these systems is ensured also in regard to COVID-19.
			(2) Did your routine system that was already in place accomodate for the COVID-19 pandemic or did you have to implement a new system?	(2) The routine system of a centralized electronic health record functioned well and smoothly, also during the COVID-19 pandemic.
			(3) Can data for secondary purposes – such as quality control, planning and policy-making – easily be extracted from these systems?	(3) Tailored aggregated datasets can be extracted easily by administrators, managers and health care staff.

			(4) What is the coverage and quality of information on medical procedures registered in the health service records?	(4) There is high coverage of registration of care provided – including diagnostic tests, treatments (surgery, drugs, other), medical devices, type of care (inpatient, ambulatory, emergency) and length of stay – in the health service records, and appropriate international classifications are used (such as International Classification of Health Interventions). Biases affecting these data are limited and known.
			(5) What is the coverage and quality of information on COVID-19 diagnoses registered in the health service records?	(5) Coverage of COVID-19 diagnoses registered in the health service records is high. For hospital discharge records ICD-10 is used to register diagnoses, and in primary health care International Classification of Primary Care is used. Biases affecting these data are limited and known.
Data sources_5	Data sources_6	Which (preventive) health programmes are implemented?	(1) What is the coverage and quality of COVID-19 testing?	(1) There is a central, national database with programme-based data on all COVID-19 testing.
			(2) How is the coverage and quality of information from the COVID-19 vaccination programme?	(2) There is a central, national database with programme-based data on all vaccinations in the vaccination programme, with full coverage.
			(3) Are adverse vaccine effects for COVID-19 registered?	(3) There is a central, national database with programme-based data on all adverse effects, including COVID-19 vaccinations.
Data sources_6	Data sources_7	Are regular health surveys conducted?	(1) Are regular health surveys/ online panels carried out in the framework of COVID-19?	(1) A (long-term) operational plan is in place, including financing from the public budget, for (regular) conducting of national health surveys. The methodology applied in these surveys is in accordance with international standards and, if applicable, international data delivery requirements. Specific efforts are made to make sure that hard-to-reach groups are adequately represented in the sample (e.g. people in long-term care facilities, non-native speakers, homeless people) also in relation to COVID-19.
			(2) Do health and statistical authorities work together on survey design, implementation and data analysis and use?	(2) Cooperation mechanisms exist (e.g. between the public health institute, statistics office and universities).
Data sources_7	Data sources_8	What data sources on health care resources exist?	(1) What data sources exist on human resources?	(1) A national human resources database tracks the number of health professionals by major professional category working in either the public or the private sector, with complete coverage. A national database tracks the annual numbers graduating from all health training institutions, with complete coverage. Each individual health care provider in the national human resources database has been assigned a unique identifier code, which stays with them for their practising career; this permits data on the same provider to be merged.
			(2) What data sources exist on facilities to address the crisis to e.g. the number of hospital beds.	(2) A national database of public and private sector health facilities is in place, with complete coverage. Each health facility has been assigned a unique identifier code that permits data on facilities to be merged.
**Data infrastructure**				
Data infrastructure_1	Data infrastructure_1	What is the status of adoption of EHR and other electronic information systems in the national HIS?	(1) Are EHR systems being used by health care facilities/providers?	(1) Health care facilities and providers only use electronic patient records; there is no parallel paper record keeping.
			(2) Is an electronic system for registration of deaths in place?	(2) An electronic system for registration of deaths is in place.
			(3) Is an electronic system in place for notification and registration of COVID-19?	(3) An electronic notification system for COVID-19 is in place, with real-time data, allowing authorities to respond immediately.
			(4) Were these electronic information systems adapted to address COVID-19? Describe the technical changes that were implemented to deal with the crisis.	(4) In case the system in place had to be adapted, such changes are clearly documented.
			(5) Can COVID-19 patients access their own data in the EHR system?	(5) Patients can access their own data, empowering them by allowing them to check these for completeness and correctness.
Data infrastructure_2	Data infrastructure_3	Are any interoperability standards in relation to COVID-19 defined and used?	(1) Are any commonly agreed interoperability requirements or standards in place for the information systems in the HIS and wider information systems on COVID-19?	(1) The standards that health care providers and facilities need to use to communicate between organizations and to report to authorities are defined.
			(2) Are any accreditation standards in place for digital services and applications that focus on ensuring interoperability with other services and applications?<br>(3) Which organizations or bodies are responsible for development of health sector and broader national standards?<br>(4) Which organizations or bodies are responsible for undertaking conformance, compliance and accreditation of products and services – including ICT – used in the health sector?	(2–4) Specific offices/agencies are in charge of defining official interoperability standards, compliance with these standards and accreditation of products and services.
			(5) What is the level of adoption of interoperability standards among existing health services and applications?	(5) An overview of the level of adoption of health care standards is available (for example, through a survey); the level of (planned) adoption is high.
			(6) Did this level change during the crisis or was the level of adoption changed due to the crisis?	(6) The level of adoption of interoperability standards was adaquate to accomodate information flows during the crisis.
Data infrastructure_3	Data infrastructure_4	Is a unique personal identification number (UPIN) in use?	(1) Is a UPIN issued at birth for each citizen?	(1) A UPIN is issued at birth for each citizen, and this is used across government services, including health services.
**Data management**				

Data management_1	Data management_1	What metadata standards are in use?	(1) Are common standards in use?<br>(2) Are these standards aligned with international standards?	(1–2) Common standards are used for metadata for official (health) statistics, which are aligned with international metadata standards.
			(3) Are the COVID-19 metadata made available?	(3) COVID-19 Metadata are structurally available for all official statistics and can be easily located and accessed by users of these statistics.
			(4) If adjustments such as standardization or weighing are used, how are these selected?	(4) In-country adjustments use transparent, well established methods. If a weighing factor is applied, methods and variables used are clearly described.
Data management_2	Data management_2	What quality control mechanisms are applied for the data sources listed above?	(1) What kind of automated and or/manual control mechanisms are built into the EHR/information systems for COVID-19 ?	(1) Automated logic checks are built into the systems/ Regular manual checks are performed according to well established and well documented protocols to check for completeness and correctness of the COVID-19 data.
			(2) Are audits performed to check the completeness and correctness of COVID-19 data?	(2) Regular audits are performed – e.g. by the health insurance company or ministry of health – to check the quality of the diagnosis-related group information submitted by health care facilities.
Data management_3	Data management_3	How can COVID-19 data sources be accessed and used for secondary purposes?	(1) Are publicly funded data sources publicly available and published as open data?	(1) Publicly funded data sources are publicly available and published as open data (provided that the necessary data protection safeguards have been taken into account).
			(2) Is there an electronic platform for exchanging COVID-19 data between (semi-) governmental organizations?	(2) An electronic data exchange platform for the safe and efficient exchange between (semi-)governmental organizations is in place. All government departments and other relevant agencies are connected to the platform.
			(3) Is a central COVID-19 data warehouse in place?	(3) An integrated COVID-19 data warehouse is operated at the national level, containing data from all data sources (both population-based and facility-based sources, including all key health programmes). The data warehouse has a user-friendly user interface, which is accessible to various user audiences and which allows for the tailored extraction of data and indicators.
			(4) Are opportunities available to link COVID-19 data sources safely at the subject level and perform comprehensive analyses – for example, through a closed controlled working environment operated by the statistical office, or through anonymization and linkage by a trusted third party.	(4) Opportunities are available to link COVID-19 data sources safely at the subject level and perform comprehensive analyses – for example, through a closed controlled working environment operated by the statistical office, or through anonymization and linkage by a trusted third party.
Data management_4	Data management_4	Are COVID-19 international data delivery requests being met?	(1) Are COVID-19 data collection methods and analytical approaches (e.g. calculation of indicators) in line with international standards and recommendations?	(1) COVID-19 data collection methods are in line with international standards and recommendations.
			(2) Is the country able to meet all COVID-19 data delivery requirements from the international organizations of which it is a member/with which it is collaborating?	(2) The country can fulfil all COVID-19 health information requests from international organizations.
			(3) How much strain is that putting on COVID-19 health system of the country?	(3) The courntry can fulfil all COVID-19 health information requests from international organizations, this is not putting any strain on the health system.
			(4) Does the country participate in international health information projects or activities regarding COVID-19?	(4) Which actors are involved in COVID-19 international projects or activities is known. Developments with regard to health information at an international level are routinely monitored and shared by experts in the HIS.
**Resources**				
Resources for data collection_1	Resources for data collection_1	Is an adequate legal framework in place for HIS data collections for COVID-19?	(1) Is there a legal basis for the COVID-19 data collections?	(1) There is a clear legal basis for the most important COVID-19 data collections.
			(2) Which data elements are defined in the law?	(2) Specific data standards (e.g. disaggregation levels and ICD-10 codes) are defined.
			(3) Is an adequate legal framework in place for linking and sharing the COVID-19 data collections?	(3) Criteria for data privacy, secondary processing, sharing of information and data linkage are specified. A legal framework that is not too restrictive is in place – i.e. one that strikes the right balance between data protection and making health data available for the public good. Accessibility of essential data sources for the most important HIS stakeholders (statistical office, ministry of health) is regulated by law.
			(4) Are requirements for COVID-19 data storage defined in the law?	(4) Requirements for COVID-19 data storage are clealry defined in the law.
			(5) Have legal aspects limited the country in the COVID-19 data collection?	(5) The legal basis that was in place did not limit data collections for COVID-19.
Resources for data collection_2	Resources for data collection_2	Are sufficient human resources available for maintaining and operating COVID-19 data collections?	(1) Do HIS stakeholders have adequate tools to maintain and operate COVID-19 data collections?	(1) HIS stakeholders have adequate tools (e.g. database and data management software) to maintain and operate COVID-19 data collections.
			(2) Do HIS stakeholders have adequate manpower to maintain and operate COVID-19 data collections?	(2) HIS stakeholders have adequate manpower, and staff turnover is limited.
			(3) Do HIS stakeholders have adequate capacity to maintain and operate COVID-19 data collections?	(3) HIS stakeholders have adequate capacity – i.e. staff with the right technical skills and expertise. Regular training is provided/funds are available for regular training.

Item ID	Item ID_WHO	Question	Probing question	Expectations
**Data analysis**				
Analysis_1	InfDisSurv_5	How is analysis of COVID-19 surveillance data performed?	(1) Which stakeholders are involved in the design and implementation of COVID-19 surveillance strategies and data analysis?	(1) Health and statistical authorities work together on the design and implementation of COVID-19 surveillance strategies and data analysis. Cooperation mechanisms exist between the public health institute, statistics office, universities and others. Examples are provided.
			(2) What tools are in use for analysis of COVID-19 data? Please provide examples.	(2) Examples are given of electronic platforms that integrate, synthesize and visualize information pertaining to COVID-19 surveillance. Computer-generated customized reports, tables, charts, maps and metadata are implemented.
			(3) Are COVID-19 data collection methods and analytical approaches (e.g. calculation of indicators) in line with international standards and recommendations? Please provide examples.	(3) COVID-19 data collection methods are in line with international standards and recommendations, and the country fulfils all health information requests from international organizations.
			(4) Is analysis of COVID-19 data conducted routinely by person and cause/risk factor/mode of transmission? Please describe specific data outputs.	(4) Data analysis is performed by person (e.g. age, sex, race), biological characteristics (e.g. immune status), acquired characteristics (e.g. marital status), activities (e.g. occupation, leisure activities, use of medications/tobacco/drugs) or the conditions in which they live (e.g. socioeconomic status, access to medical care), according to documented SOPs. Standardized rates of disease are calculated by person. Tables and graphs, along with interpretations, are produced. Examples are provided.
			(5) Is analysis of COVID-19 surveillance data routinely conducted by place? Please describe specific data outputs.	(5) Occurrence of COVID-19 is described by relevant geographical location (i.e. place of diagnosis or report, birthplace, site of employment, school district, hospital unit or recent travel destinations) according to documented SOPs. Tables and maps, along with interpretations, are produced. Examples are provided.
**Indicators**				
Analysis_2	Analysis_1	Is a core set of health indicators defined for COVID-19 and its wider effects?	(1) How were the core COVID-19 indicators selected?	(1) COVID-19 core indicators were transparently identified for national and subnational levels. Selection of indicators is also informed by international indicator sets.
			(2) Which categories does the COVID-19 indicator set cover?	(2) Indicators cover all categories of health indicators, such as determinants of health; health system inputs, outputs and outcomes (health systems performance assessment); health status; and health inequalities. If possible, the set includes relevant indicators from other policy sectors (e.g. social affairs, education).

			(3) How are the indicators defined and calculated?	(3) Indicator definitions exist and the method for their calculation is documented. If applicable, the numerator and denominator of the indicators are clearly defined.
			(4) Are metadata available and harmonized within the country and across countries?	(4) Regularly updated metadata exist for each indicator and are publicly available. Metadata include the categories definition, calculation/method, available dimensions/subgroups (e.g. age, gender, geographical information, nationality, migration, social status – e.g. education, employment status, income), rationale and data sources.
Analysis_3	Analysis_2	What kind of analyses are performed on the core indicators in the framework of COVID-19 and its wider effects?	(1) Are subnational comparisons made?	(1) Core indicators can be broken down according to relevant subnational entities (e.g. regions, municipalities). Subnational disaggregations are produced regularly.
			(2) Are international comparisons made?	(2) Definitions of national core indicators are aligned with international definitions to allow international comparisons; these are produced regularly. If different definitions and/or data sources are used for national indicator values and for international comparisons, the reasons for this are clearly explained in the indicator metadata.
			(3) Are future projections made?	(3) Periodic population projections are made. These are used to make demographic projections for key indicators. If adequate trend data are available, combined demographic and epidemiological projections are made for key indicators.
			(4) Are comparisons between subgroups made/are analyses of health inequalities performed?	(4) Data for the core indicators can be disaggregated according to age, sex, socioeconomic status and other relevant stratifiers (e.g. ethnicity). Disaggregated indicator values are produced regularly.
Analysis_4	Analysis_3	Are these COVID-19 core indicators reported/published regularly?	(1) How and how often are they reported/published/updated?	(1) Core indicators are regularly reported in standardized tables, in health reports and in basic tools for spatial comparisons and comparisons over time.
			(2) What is the frequency at which data for the core indicators become available?	(2) The datasets used to calculate the core indicators are updated regularly, and the frequency of these updates is in line with policy needs.
			(3) Are regular publication dates/periods available for each indicator?	(3) A publication schedule is available.
**Big Data and Artificial Intelligence (AI)**				
Analysis_5	Analysis_6	Is the country investing in Big Data and Artificial Intelligence (AI) research and development related to COVID-19?	(1) Is a Big Data/AI strategy related to (public) health for COVID-19 related issues in place?	(1) A strategy for Big Data/AI, either standalone or as part of the digital health/eHealth strategy or another national digital health initiative, is in place.
			(2) Is action on Big Data/AI included in the national budget?	(2) The national health and/or research budget includes dedicated funds for action on Big Data/AI.

			(3) Has the Big Data/AI strategy changed to address the COVID-19 crisis?	(3) Innovative mechanisms were set up to address issues such as contact tracing, vaccine registrtaion, apps etc.
			(4) Are ethical Big Data/AI requirements, standards and best practices listed and respected?	(4) A set of ethical principles is defined, together with policies and regulations. Best practices are actively shared.
			(5) Are infrastructure requirements for Big Data/AI and data science defined?	(5) The following infrastructure requirements are clearly defined: computing capacity, storage capacity, networking infrastructure, security policies.
			(6) Is the country working on transforming the health information workforce to be fit for the new Big Data/AI era?	(6) The country has a plan to train health information professionals in data science and Big Data/AI, including adequate funding.
**Resources**				
Analysis_6	Analysis_7	Do HIS stakeholders have adequate resources to analyse and report on COVID-19 indicators regularly?	(1) Do HIS stakeholders have adequate tools for regular analysis and publication of COVID-19 indicators?	(1) HIS stakeholders have adequate tools for analysis (e.g. computers, servers, analysis software) and publication (e.g. module for interactive dashboard).
			(2) Do HIS stakeholders have adequate manpower for regular analysis and publication of COVID-19 indicators?	(2) HIS stakeholders have adequate manpower, and staff turnover is limited.
			(3) Do HIS stakeholders have adequate capacity for regular analysis and publication of COVID-19 indicators?	(3) HIS stakeholders have adequate capacity – i.e. staff with the right skills and expertise (such as statisticians, epidemiologists, geographical information system (GIS) experts, data visualization experts, communication experts). A multidisciplinary team works on publication of the core indicators. Regular training is provided/funds are available for regular training on analysis skills.

Item ID	Item ID_WHO	Question	Probing question	Expectations
**Health reporting**				
Health reporting_1	Health reporting_1	Is there capacity for reporting and publication of COVID-19 health reports and surveillance information?	(1) Are such health reports being produced on a regular basis, and by whom? A health report can either be a more traditional report in paper/PDF format, or a web-based report/website.	(1) Regular surveillance reports are produced by the national public health agency or comparable institutions, independent of the ministry of health. Scientific standards and common transparency requirements are followed in the production of the reports.
			(2) Is information from the COVID-19 surveillance system available for population health monitoring?	(2) Information from COVID-19 surveillance is readily available for use in population health reports, where it can be placed in a broader context.
			(3) How comprehensive are these reports?	(3) Epidemiological reports are written in easy-to-read language, use a combination of texts and informative visualizations and include key messages, analysis outputs, interpretation of results and options for actions (i.e. assessment of preventive measures).
			(4) Are (COVID-19) foresight and scenario exercises included in the reporting efforts?	(4) Foresight and scenario exercises are performed to inform long-term strategic health policy-making.
			(5) What format do the health reports use?	(5) Regular health reports use standard reporting formats and tools, preferably publicly available web-based reports that use interactive visualization tools that allow generation of tables, graphs/charts, maps and infographics or COVID-19 dashboards. It is possible to download the visualizations and the data on which they are based. Tailored summaries/factsheets are available for different target audiences.
			(6) Are these reports publicly available?	(6) Health reports or surveillance reports are publicly available and readily accessible.
			(7) What kind of communication and dissemination strategies are used for these reports? Are infodemic management tools used?	(7) Comprehensive communication and dissemination strategies for COVID-19 are in place, including mass media, social media, online health (information) platforms, newsletters, email messages, presentations and lectures. Active after-care is also part of the communication and dissemination strategy, including follow-up on social media. Experts talking to the mass media have received relevant training.
**Policy integration**				
Health reporting_2	Health reporting_2	What mechanisms exist for using COVID-19 health and surveillance reports in the policy-making process?	(1) What is the mechanism for using COVID-19 health and surveillance reports in the health policy-making process?	(1) There is a formal, public and transparent procedure for using COVID-19 health and surveillance reports in the policy-making process. Parliament is informed by the ministry of health when formal health reports are published.
			(2) Are the COVID-19 health and surveillance reports used to inform intersectoral policy-making, and do other policy sectors also include information on health in their reporting efforts/use health information for informing their policies (a Health in all policies approach)?	(2) An intersectoral governmental body that discusses (how to use) the COVID-19 health and surveillance reports is in place, and its decisions are formally and publicly reported. Health is a standard dimension in reports of other policy sectors.
			(3) Is it known to what extent policy-makers and other users (such as media, patient organizations, NGOs, professional organizations) actually use the reports?	(3) User surveys are conducted regularly. Website statistics are monitored and analysed regularly. Reports about the results of the user surveys and website statistics are publicly available.
Health reporting_3	Health reporting_3	Are COVID-19 health reports produced at the health care facility and provider levels?	(1) Do managers and medical staff use health reports to monitor and improve performance (e.g. quality control, patient safety)?	(1) Managers and medical staff regularly use health reports to monitor and improve performance. Such reports are discussed jointly (e.g. at the department or team level) and ways to improve are decided together. There is an open attitude among health care staff towards measuring and monitoring performance. Health care staff feel safe to discuss (suboptimal) quality of care and performance.
			(2) What kind of indicators are used for these reports?	(2) Indicators that are acknowledged by (international) peers as valid and useful are used. Indicators used include patient-reported outcomes (PROMs) and patient-reported experiences (PREMs).
**Resources**				
Health reporting_4	Health reporting_4	Do HIS stakeholders have adequate resources for producing and publishing regular COVID-19 health reports?	(1) Do HIS stakeholders have access to adequate tools for health reporting?	(1) HIS stakeholders have access to adequate tools for producing health reports (e.g. quality criteria/toolkit, evidence resources) and publishing health reports (e.g. software for creating interactive graphs and options for integrating videos in online reports).
			(2) Do HIS stakeholders have adequate manpower for producing and publishing regular health reports?	(2) HIS stakeholders have adequate manpower, and staff turnover is limited.

(3) Do HIS stakeholders have adequate capacity for producing and publishing regular health reports?	(3) HIS stakeholders have adequate capacity, i.e. staff with the right skills and expertise (such as statisticians, epidemiologists, GIS experts, data visualization experts, writers/editors, communication experts). A multidisciplinary team works on producing the health reports. Regular training is provided/funds are available for regular training on reporting skills.

Item ID	Item ID_WHO	Question	Probing question	Expectations
**Information and knowledge products**				
Knowledge translation_1	Knowledge translation_1	Do relevant stakeholders know which COVID-19 information and knowledge products are available, and are they able to use them efficiently?	(1) Are information products regularly demanded by users like senior managers and policy-makers?	(1) The regular information and knowledge products produced within the HIS and their publication schedules are well known by policy-makers, senior managers and other actors such as media representatives. Senior managers and policy-makers demand complete, timely, accurate, relevant and validated HIS information, and know how to interpret and use it.
			(2) Are support mechanisms available to train relevant actors on how to interpret and use the products?	(2) Training or information courses on the products and their use are offered regularly.
			(3) Does the COVID-19 information produced within the HIS meet the needs of the policy-makers?	(3) Regular exchange sessions take place to identify the information needs and to assess the timeliness and usefulness of the formats with policy-makers and other relevant users. The outcomes of these sessions and implemented changes are documented and reported. Exchange and integrated knowledge translation approaches are applied to make sure that information and knowledge produced meet the needs of policy-makers.
			(4) What kind of communication mechanisms are in place if there are questions or ad hoc requests for this type of information?	(4) A rapid response team/mechanism is in place to respond quickly to ad hoc questions (e.g. when the ministry of health is looking for health information to answer questions from parliament).<br>After-care is a structural element in the communication and dissemination plans for health information and knowledge products.<br>A regularly conducted user survey is applied to identify the usability of health information and knowledge products.
Knowledge translation_2	Knowledge translation_3	What kind of knowledge translation tools and mechanisms are used?	(1) Are specific tools to stimulate uptake of information and knowledge in policy-making used?	(1) Producers of reports use tools specifically aimed at stimulating uptake of information and knowledge in policy-making, such as policy briefs and policy dialogues.
			(2) Alongside the more traditional *push* and *pull* mechanisms, are *exchange* and *integrated* approaches also applied for knowledge translation?	(2) Exchange and integrated approaches for knowledge translation are applied. In exchange approaches, information analysts and relevant users of the HIS work in partnership, often facilitated by knowledge brokers, to collect the necessary evidence. In integrated approaches, a knowledge translation infrastructure is institutionalized and represents clear objectives for action, regular assessments of the relevance of its efforts and incorporation of elements of push, pull or exchange efforts.
			(3) To what extent are the applied knowledge translation tools and mechanisms institutionalized?	(3) The applied knowledge translation tools and mechanisms are institutionalized: they a structural element of the health policy-making process.
**Resources**				
Knowledge translation_3	Knowledge translation_4	Do HIS stakeholders have adequate resources for COVID-19 knowledge translation?	(1) Do HIS stakeholders have adequate manpower for knowledge translation?	(1) HIS stakeholders have adequate manpower for knowledge translation.

(2) Do HIS stakeholders have adequate capacity for knowledge translation?	(2) HIS stakeholders have adequate capacity. Staff have been trained in knowledge translation concepts, tools and skills, and adequate budget id available for training to keep staff capacity up to date.
(3) Was the distribution of resources for knowledge translation changed during the COVID-19 pandemic?	(3) The distribution of resources was adaquate.

Item ID	Item ID_WHO	Question	Probing question	Expectations
**Legislation**				
Governance and resources_1	Governance and resources_1	Is the legislation providing the legal framework for the COVID-19 HIS up to date?	(1) Does the country have health legislation?	(1) The legislation exists and is enforced. The legislation defines the tasks of the public health authority. The legislation covers WHO's essential public health operations. Ideally, this legal framework should also cover an evidence-informed policy cycle.
			(2) Does the public health service law include population health monitoring and maintenance of an HIS as a mandatory task of the public health authority?	(2) Population health monitoring and the maintenance of the HIS are part of the legislation.
			(3) Does the public health service law also define tasks that cover the whole policy cycle?	(3) The public health service law defines tasks and roles that cover the whole policy cycle (problem definition, agenda setting, policy formulation, decision-making, policy implementation, policy evaluation).
			(4) Does the country have electronic HIS legislation that governs how health information is stored, accessed and shared across geographical and health sector boundaries?	(4) A legislative framework for the electronic HIS is in place. The legislation defines which (international) classifications must be used (e.g. International Statistical Classification of Diseases and Related Health Problems, 10th revision (ICD-10), System of Health Accounts). National interoperability standards and other requirements are developed. Compliance, conformance and accreditation of electronic health information products and services are defined and implemented. The legislation ensures equal access for all citizens to their own health data.
			(5) What policies exist to stimulate and manage innovations in the field of electronic HIS, such as who is responsible for introducing change and innovation, how risks are managed and how to evaluate appropriateness, feasibility and utility?	(5) Policies are in place that stimulate and manage innovations in the field of electronic HIS. Best practices and evaluation reports are collected in a central place and made publicly available.
Governance and resources_2	InfDisSurv_10	What legal and regulatory framework supports implementation of COVID-19 and other infectious disease surveillance?	(1) Have infectious diseases and pathogens been prioritized for surveillance? When was the list of priority diseases last updated?	(1) Evidence exists of prioritization of infectious diseases and pathogens for surveillance. A list of priority diseases and case definitions is available. The list is updated as necessary and includes epidemic-prone diseases like cholera, diarrhoea with blood, measles, meningitis, plague, viral haemorrhagic fevers, yellow fever, severe acute respiratory syndrome and diseases targeted for eradication/elimination.
			(2) Are objectives for disease surveillance clearly stated and documented?	(2) Clear objectives for surveillance of infectious diseases and pathogens have been defined (as evidenced by relevant documentation).
			(3) What is the legal and regulatory framework for implementation of infectious disease/pathogen surveillance activities?	(3) A national legal framework (laws and regulations) for infectious disease/pathogen surveillance is available and updated regularly.
			(4) Were there any issues in performing surveillance for COVID-19 due to legal constrains?	(4) COVID-19 surveillance was not hampered by the regulaory framework in place.
			(5) Is the legal and regulatory framework for implementation of infectious disease/pathogen surveillance activities in line with relevant international standards?	(5) A national legal framework (laws and regulations) for infectious disease/pathogen surveillance is in line with relevant international standards.
			(6) Is there a memorandum of understanding or other agreement between public health and security authority entities at the national level with regard to detection, investigation and response to public health emergencies?	(6) SOPs or emergency response plans that include security authorities are in place to link public health and security authorities (e.g. law enforcement, border control, customs) during a suspected or confirmed public health emergency if required.
**Policies, planning and evaluation**				

Governance and resources_3	Governance and resources_2	Does the country have a comprehensive HIS strategic plan?	(1) Is an HIS strategic plan to monitor the wider effects of COVID-19 in place? (shorter term and longer term)	(1) A formal comprehensive HIS strategic plan to monitor the wider effects of COVID-19 is in place, either as a standalone strategy or as a clearly discernible element of a wider health/health systems strategy. The most important HIS stakeholders, including other relevant ministries beside the ministry of health, were involved in the development of the strategic plan and are involved in its monitoring and evaluation. This HIS strategic plan defines an HIS vision and clear goals.
			(2) Does the HIS strategic plan include aspects of electronic health information services, or does the country have a separate strategy for this?	(2) The HIS strategic plan includes aspects of electronic health information services. If this is not the case, a separate strategic plan/policy for electronic health information services exists.
			(3) What kind of monitoring and evaluation mechanisms for the strategic plan are in place?	(3) The strategic plan defines mechanisms for monitoring progress towards the goals (e.g. what indicators will be used, who will collect data for these indicators and report on them, how often progress will be assessed).
			(4) How well integrated is the HIS strategic plan between the national, state, regional and local levels (and different policy sectors)?	(4) The strategic plan lays out how cooperation and integration of work between different levels is performed.
Governance and resources_4	InfDisSurv_12	How is the COVID-19 surveillance strategy and its coordination conducted?	(1) Is there a monitoring and evaluation system?	(1) All reporting sites are routinely monitored. The infectious disease/pathogen surveillance system has been evaluated in the last five years.
			(2) Are roles and responsibilities well defined and documented?	(2) Roles and responsibilities are well defined and documented at each level of the infectious disease/pathogen surveillance system.
			(3) Is a coordinating body in place?	(3) A surveillance unit is in place at the national level for coordination of infectious disease/pathogen surveillance activities.
			(4) Are any mechanisms in place to link public health and security authorities during a suspected or confirmed public health emergency?	(4) SOPs or emergency response plans that include security authorities are in place. Mechanisms exist that link public health and security authorities (e.g. law enforcement, border control, customs) during a suspected or confirmed public health emergency if required. Information reports are shared regularly with security authorities.
Governance and resources_5	InfDisSurv_11	Is a plan of action to enhance the infectious disease/pathogen surveillance system in place?	(1) Are strategic and operational plans for implementing and strengthening infectious disease/pathogen surveillance in place?	(1) Strategic and operational plans for implementing and strengthening communicable disease surveillance and response systems are in place. These are updated every 3–5 years for strategic plans and annually for operational plans.
			(2) How many activities have been implemented according to the plans?	(2) Annual activity reports are available. Most activities have been implemented according to the plans.
Governance and resources_6	Governance and resources_7	What is the status of the ICT infrastructure for COVID-19 data flows in the national HIS?	(1) Is the availability of hardware sufficient?<br>(2) Is the availability of software sufficient?<br>(3) Are enough skilled ICT staff available?	(1–3) Adequate ICT infrastructure (e.g. computers, internet access, servers) and adequate ICT support are in place at the national level, at relevant subnational levels and at the hospital/provider level.
			(4) What is the capacity and coverage of data connectivity and networking across the country, including metropolitan, regional, rural and remote areas?	(4) Sufficiently fast internet is available throughout the country, including in remote rural areas.
			(5) Did the ICT infrastructure accomodate COVID-19 data flows?	(5) The ICT structure that was in place accomodated the data flows regarding COVID-19 data.
			(6) What is the level of ICT skills of people working in the HIS? Note: this relates to people other than dedicated ICT staff – e.g. civil servants, scientific staff, medical staff.	(6) People working in the various institutions with a role in the HIS (e.g. ministry of health, public health institute, statistical office, health care facilities) have adequate ICT skills and access to training if needed.
Governance and resources_7	InfDisSurv_13	Are regular advanced training options available for people with roles in infectious disease/pathogen surveillance?	(1) What proportion of surveillance units has completed training modules?	(1) A high proportion of surveillance units complete training modules every year.
			(2) What proportion of staff/health care workers is trained on surveillance of infectious diseases?	(2) A high proportion of staff/health care workers completes training modules on infectious disease/pathogen surveillance every year.
			(3) What proportion of epidemiologists is trained on infectious disease/pathogen surveillance in each district?	(3) All epidemiologists are trained on infectious disease/pathogen surveillance.

			(4) What proportion of staff/health care workers has received a refresher course on surveillance of infectious diseases?	(4) A high proportion of staff/health care workers has undergone refresher training modules on infectious disease/pathogen surveillance in the past two years.
			(5) Has training been conducted jointly (at an intermediate (regional) or national level) with both public health and security authorities on topics related to infectious disease/pathogen information sharing and joint investigations of public health emergencies?	(5) The country has organized and conducted training jointly with public health and security authorities on topics related to infectious disease/pathogen information sharing and joint investigations of and responses to public health emergencies.
Governance and resources_8	InfDisSurv_14	Have the attributes of the COVID-19 surveillance system been evaluated?	(1) What is the frequency of surveillance reports (immediate, weekly, monthly)? Please provide examples.	(1) The frequency of notifications is appropriate, based on the surveillance objectives and the epidemiology of the disease. Examples are provided.
			(2) How is the absence of underreporting (external completeness) of the COVID-19 surveillance system evaluated?	(2) Evaluation of external completeness is planned and implemented at least for priority diseases requiring a high level of completeness. Possible methods for evaluation of external completeness include reviews of a sample of all surveillance data, reviews of medical records and capture–recapture studies. Underreporting has triggered corrective actions (documented).
			(3) What procedures are in place to assess internal completeness? What is the proportion of surveillance reports with no missing required information (variables)?	(3) A high proportion of completeness of variables is reported. (1: highly adequate if 90% or more; 2: adequate if 75–89%; 3: present but not adequate if 25–74%; 4: not adequate at all if less than 25%.) A mechanism is in place to monitor internal completeness. Low internal completeness has triggered corrective actions (documented).
			(4) How representative is the COVID -19 surveillance system? What is the geographical coverage? Do all clinical settings participate? Is the prevalence of urban versus rural reporting sites evenly distributed? Are minority populations reached by the surveillance system?	(4) COVID-19 cases notified are representative of the population under surveillance for a defined geographical area, based on surveillance objectives.
			(5) Is the external validity of the data generated by any infectious disease surveillance system evaluated? What methods are used? Please provide examples.	(5) Designated staff perform validity checks. Possible external data sources that can be used to validate surveillance data are listed. Retrospective (i.e. concordance evaluation with medical records) or prospective evaluation methods are used. Methodology for validity checks and results (e.g. duplicate records, case misclassification, invalid data collection formats) are described thoroughly. Examples are provided. The procedures are documented with relevant protocols and SOPs.
			(6) Is the internal validity of the data generated evaluated? What methods are used? Please provide examples.	(6) Validity checks such as cross-tabulations and distribution analysis of variables are routinely performed to identify outliers and inconsistent values. A descriptive analysis of data is compared to expected values, based on available background surveillance data, to identify any deviations. Examples are provided. The procedures are documented with relevant protocols and SOPs.
			(7) Are COVID-19 data flow and operating procedures sufficiently clear to all relevant stakeholders?	(7) Implementers and users of the system rate the procedures as sufficiently simple and clear. The amount and type of data collected, managed and analysed is appropriate. The number of organizations involved in receiving case reports from a surveillance unit and the time spent on system maintenance is kept to a minimum while still meeting surveillance objectives.
			(8) Is the COVID-19 surveillance system able to collect, manage and provide data properly and consistently over time without failure?	(8) Data and methodology are consistent over time and available when needed. The system is fully operational at all times. Dedicated resources (human and material) for running and maintaining the surveillance activities are secured in the long term.
			(9) Has the usefulness of the COVID-19 data generated by the surveillance system been evaluated? What methods were used? Please provide examples.	(9) An inventory of actions taken as a result of information generated by the COVID-19 surveillance system has been made. Usage of the system is documented (users of information, actions). Examples of actions are given and might include timely response to health hazards, informing target groups for vaccination, hypotheses to stimulate research, hospital bed capacity increases.
